# A Case Series of Plexiform Neurofibroma: The Unusual Presentations and Surgical Challenges

**DOI:** 10.7759/cureus.23141

**Published:** 2022-03-14

**Authors:** Hui Yuan Lam, Shawaltul Akhma Bt Harun Nor Rashid

**Affiliations:** 1 Reconstructive Science Unit, Hospital Universiti Sains Malaysia, Kelantan, MYS; 2 Plastic and Reconstructive Surgery, Hospital Raja Perempuan Zainab II, Kelantan, MYS

**Keywords:** autosomal dominant neurocutaneous disorder, von recklinghausen disease, neurofibromatosis, isolated, plexiform neurofibromatosis

## Abstract

Neurofibromatosis type one (NF-1) is an autosomal dominant neurocutaneous disorder also known as Von Recklinghausen disease. Plexiform neurofibroma is a rare kind of NF-1 where the neurofibroma originates from nerve sheath cells or subcutaneous peripheral nerves. It is pathognomonic of NF-1, and isolated occurrence is relatively rare. We reported three cases of solitary plexiform neurofibromas with an unusual presentation. We have two cases of spontaneous bleeding isolated plexiform neurofibroma that have never been reported in the literature. Neither one of them showed signs and symptoms associated with the neurofibromatosis spectrum. This unusual presentation poses substantial challenges in diagnosis and management.

## Introduction

Neurofibromatosis type one (NF-1) is a benign tumor of neural origin classified as a cutaneous or subcutaneous variant (99% of incidence) or rarely as a plexiform variant [1]. A macroscopic examination of plexiform neurofibroma reveals that a substantial nerve segment has been affected. This lobulated nerve growth gives the appearance of a "bag of worms"-like feature [2]. Unlike the other types of neurofibromas, this rare benign tumor has the potential to become malignant. Isolated plexiform neurofibroma not associated with NF-1 is not commonly reported in the literature. Hence, its atypical presentation always makes it a diagnosis challenge and it is often misdiagnosed as a vascular anomaly. Two of our cases presented as bleeding huge mass that masked the diagnosis of isolated plexiform neurofibroma.

## Case presentation

Case 1

**Figure 1:**
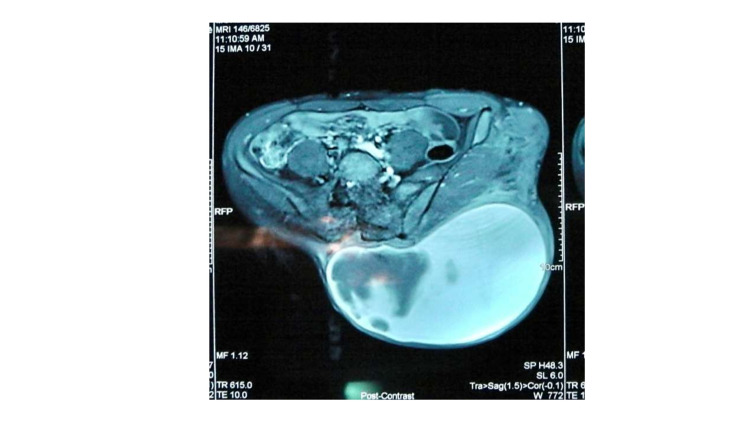
The MRI shows large heterogenous and expanded hematoma at the left gluteal region.

**Figure 2:**
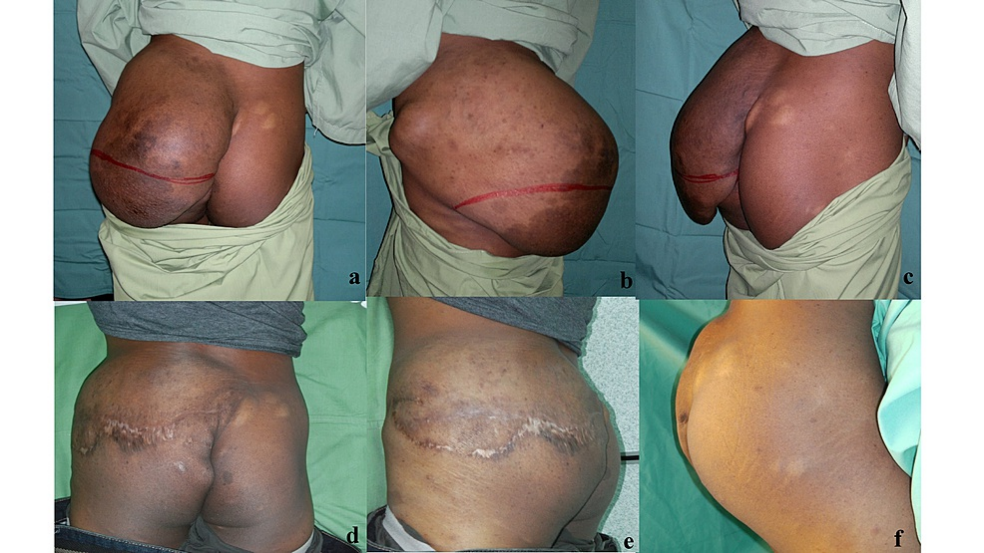
Initial presentation with different views: posterior view, left lateral view, and right lateral view (a-c). Post serial excision photos taken in different views: posterior view, lateral view, and medial view (d-f).

A 27-year-old gentleman is a known case of plexiform neurofibromatosis. He had an excision for his left gluteal neurofibroma by our team. He was well until he presented to the emergency department (ED) with a painful mass over the left gluteal region. Further history revealed a trivial fall in a sitting position two days ago. However, the swelling over the left gluteal increased tremendously, restricting his movement due to severe pain. Based on the acute event, he was initially referred to the surgical team and treated as a bleeding hemangioma. There was a huge mass with an estimated size of 20 cm x 16 cm, located at his left gluteal region extending up the left flank, which was soft in consistency but non-pulsatile. After an ultrasound revealed no signs of hemangioma, he was referred to our team, with the diagnosis pointing to an acute incident of bleeding plexiform neurofibroma. We managed to treat the swelling by compression and optimize his hemoglobin level due to the critical blood loss event. He was discharged home after 10 days of observation. After the hematoma had become well organized, he was scheduled for elective removal. However, he returned to us one week after discharge as there was an ulcer in the center of the hematoma. To shrink the bulk, he underwent serial excision. He was scheduled for another excision to achieve his bilateral gluteal balance volume. The patient was pleased with the result because he could sit comfortably while still wearing his regular clothes (Figures 1-2).

Case 2

**Figure 3:**
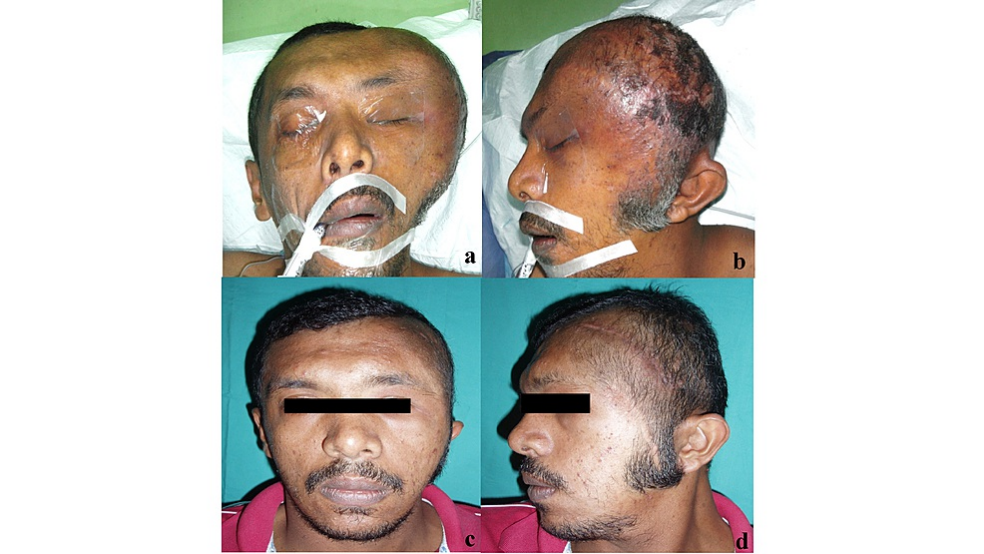
Initial presentation of the spontaneous bleeding plexiform neurofibromatosis with the remarkable size of mass over his left temporal region ( a. frontal view, b. lateral view). Photo taken after two serial excisions (c. frontal view, d. lateral view).

A 35-year-old gentleman had serial excisions for his left temporal plexiform neurofibroma over the left temporal region a few years back. Post excision, the wound was well healed, and the patient was discharged well. He came to casualty because of sudden episodes of painful swelling over the left temporal region. There was no history of preceding trauma or injury associated with the lesion. CT angiography showed a heterogeneously hyperdense lesion measuring 12.8 cm x 6.4 cm x 13.2 cm with intralesional vascularity. Given the expansion of the hematoma with neurological symptoms of vomiting, he underwent emergency surgery for exploration and control of bleeding. He had one liter of hematoma evacuated from the surrounding neurofibroma. No recurrent swelling was observed during his follow-up (Figure 3).

Case 3

**Figure 4:**
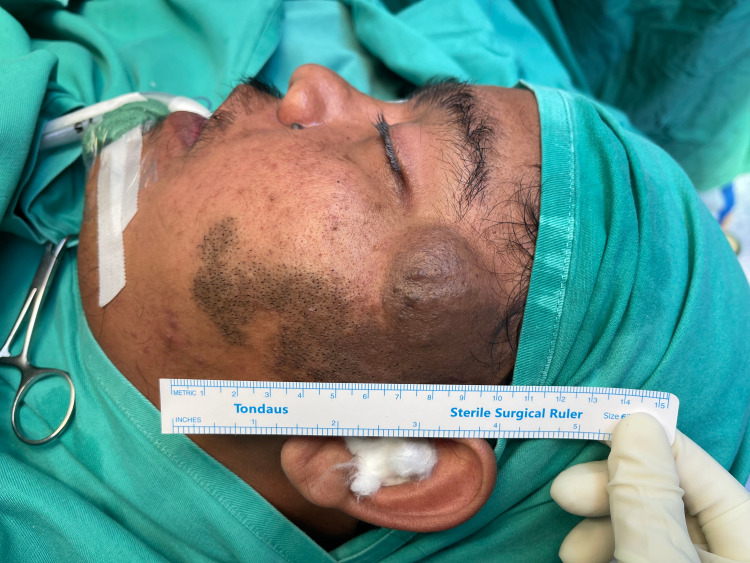
Isolated plexiform neurofibroma over the left temporal region extends to the left mandibular region.

A 30-year-old gentleman presented with a painless swelling over the left temporal region, which had been increasing in size for two years. He had no unusual medical history or family history of neurofibroma. A 10 cm x 5 cm hyperpigmented lesion in the left lateral canthus and the temporal area extends to the parotid region, noted on physical examination. It was soft in consistency, non-pulsatile, and non-mobile. There was no sign of lymphadenopathy or neuropathy on head and neck examination. Neither café-au-lait lesions were not found in the body. A clinical diagnosis of isolated plexiform neurofibroma was made. He underwent his first serial excision and a 5 cm x 5 cm lesion was removed. His wound healed with no complications in his last follow-up. We planned for his subsequent serial excision six months later (Figure 4).

## Discussion

The differential diagnosis for a sudden episode of swelling in our cases can be comprehensive. This includes abscess, hematoma, arteriovenous malformation, or malignancy. When there is no evidence of NF-1, the diagnosis of plexiform neurofibroma is rarely considered in the differential diagnosis.

Plexiform neurofibroma is pathognomonic for NF-1, which can cause significant disfigurement and functional limitation. It is typically formed in early childhood and becomes prominent as a cutaneous neurofibroma once fully grown and developed [3]. Plexiform neurofibromas have different sizes that usually develop along a large nerve trunk or any region with more fatty tissue deposition [4]. The involved nerve may be thickened and expanded, causing the surrounding tissue hypertrophy. This can result in compression to neighbor tissue structures, including the spinal cord [5].

Plexiform neurofibroma can arise from a cranial nerve. Trigeminal plexiform neurofibroma is the most common cranial nerve being reported. The lesion tends to infiltrate into the orbit, causing significant proptosis and facial disfigurement as the lesion grows into the eyelid [6]. Elephantiasis neuromata is a condition in which there is the involvement of plexiform neurofibromas to the entire extremity. The lesion follows the axis of a nervous tracking lower limb and appears hyperpigmented with loose redundant skin that can limit the limb's motor function [3].

Plexiform neurofibroma can also involve deep tissue or internal without evidence of superficial extension, including the gastrointestinal tract or urogenital area. Tamer et al. noticed that internal neurofibromas occur earlier in females, increasing during adolescence [7].

Plexiform neurofibromas are strongly associated with NF-1. However, a few cases of isolated plexiform neurofibromas of the oropharynx, tongue, orbit, the tip of the nose, and palm have been reported [8-10]. However, we have two unusual cases of isolated plexiform neurofibroma presenting with an acute bleeding episode leading to a massive mass mimicking a vascular lesion. This type of clinical presentation has not been reported in the literature.

Surgical resection is the treatment method, but it is difficult because it may include a large number of nerve branches. However, invasive plexiform neurofibroma resection and debulking are linked to a high recurrence rate. Complete resections had a recurrence rate of 20%, but incomplete resections had up to 45% reported in one case series [11]. The vascularity of these tumors, as well as their aberrant tendency to bleed, is one of the main constraints. Because of the fragile nature of the neo-vessels, these tumors bleed excessively after surgery [12]. Spontaneous bleeding is rarely linked to NF-1. It is attributed to the friable vasculature caused by arterial dysplasia or neurofibroma vascular invasion. The majority of reported cases have been linked to gastrointestinal and intrathoracic neurofibroma. The most prevalent cause of blood loss in the gastrointestinal system is caused by mucosal hypervascular plexiform neurofibroma [13].

However, massive bleeding from isolated plexiform neurofibroma is rarely reported. The optimal timing for surgery is crucial to prevent progression into hypovolemic shock. When external compression can stop the bleeding, the organized hematoma will function as a tamponade to prevent further bleeding. Once the hematoma shrinks in size, the lesion will be easier to identify and resected. However, immediate surgical intervention is needed to relieve the compression symptoms from the bleeding plexiform neurofibroma to the surrounding structure. The strategy adopted to manage our isolated plexiform neurofibroma patients is always staged excision, knowing that such a lesion has a high possibility of bleeding.

## Conclusions

Plexiform neurofibromas provide strong evidence for NF-1 diagnosis, but they might be seen as an isolated lesion without cardinal symptoms. Despite their benign appearance, plexiform neurofibromas can induce discomfort, deformity, and functional limitations, as well as spontaneous bleeding, and can be present without the clinical signs of type one neurofibroma. A precise diagnosis is required to ensure optimal planning and to anticipate difficulties caused by the underlying aberrant tissue architecture. The timing of surgical intervention is critical to preventing severe bleeding, which leads to a high illness fatality rate.
